# Effect of Patient-Specific Coronary Flow Reserve Values on the Accuracy of MRI-Based Virtual Fractional Flow Reserve

**DOI:** 10.3389/fcvm.2021.663767

**Published:** 2021-07-02

**Authors:** Jackson Hair, Lucas Timmins, Retta El Sayed, Habib Samady, John Oshinski

**Affiliations:** ^1^Department of Radiology and Imaging Sciences, Emory University School of Medicine, Atlanta, GA, United States; ^2^Department of Biomedical Engineering, Georgia Institute of Technology and Emory University, Atlanta, GA, United States; ^3^Department of Biomedical Engineering, University of Utah, Salt Lake City, UT, United States; ^4^Scientific Computing and Imaging Institute, University of Utah, Salt Lake City, UT, United States; ^5^Division of Cardiology, Department of Medicine, Emory School of Medicine, Atlanta, GA, United States

**Keywords:** CFR, FFR, MRI, CFD, vFFR

## Abstract

The purpose of this study is to investigate the effect of varying coronary flow reserve (CFR) values on the calculation of computationally-derived fractional flow reserve (FFR). CFR reflects both vessel resistance due to an epicardial stenosis, and resistance in the distal microvascular tissue. Patients may have a wide range of CFR related to the tissue substrate that is independent of epicardial stenosis levels. Most computationally based virtual FFR values such as FFR_CT_ do not measure patient specific CFR values but use a population-average value to create hyperemic flow conditions. In this study, a coronary arterial computational geometry was constructed using magnetic resonance angiography (MRA) data acquired in a patient with moderate CAD. Coronary flow waveforms under rest and stress conditions were acquired in 13 patients with phase-contrast magnetic resonance (PCMR) to calculate CFR, and these flow waveforms and CFR values were applied as inlet flow boundary conditions to determine FFR based on computational fluid dynamics (CFD) simulations. The stress flow waveform gave a measure of the functional significance of the vessel when evaluated with the physiologically-accurate behavior with the patient-specific CFR. The resting flow waveform was then scaled by a series of CFR values determined in the 13 patients to simulate how hyperemic flow and CFR affects FFR values. We found that FFR values calculated using non–patient-specific CFR values did not accurately predict those calculated with the true hyperemic flow waveform. This indicates that both patient-specific anatomic and flow information are required to accurately non-invasively assess the functional significance of coronary lesions.

## Introduction

Coronary artery disease (CAD) is responsible for half of all deaths attributed to cardiovascular disease, making it a leading cause of death globally (1, 2). Not all patients with CAD are at risk for adverse events, and it is therefore important to be able to correctly identify which patients would benefit from percutaneous coronary intervention (PCI). The gold standard for making this determination is through assessment of the functional significance of the stenosis by fractional flow reserve (FFR), which is approximated in the catheterization lab as the ratio of the pressure distal to a lesion over the proximal pressure (3). The pressure is expected to scale linearly with the flow rate if the resistance is constant and minimized, which is achieved through induction of hyperemia using an injected vasodilator—such as adenosine—and averaging measurements across multiple cardiac cycles (3). Therefore, this pressure ratio provides an approximation for the flow reduction caused by the plaque. Several studies have shown the benefits and efficacy of FFR in deciding who would benefit from PCI (4, 5), and FFR is the only diagnostic method for guiding coronary intervention that has shown any benefit to patient outcomes to date. Unfortunately, despite its proven efficacy, survey data from coronary interventions of intermediate stenoses have shown that FFR is used in only 6.1% of patients while 73.6% of patients are evaluated with angiography alone (6, 7). This underutilization can be attributed largely to the extra time and cost of the pressure wire, as well as the small but non-negligible risk to the patient. Therefore, there have been considerable efforts in recent years to develop non-invasive alternative methods of determining FFR.

**Figure 1:**
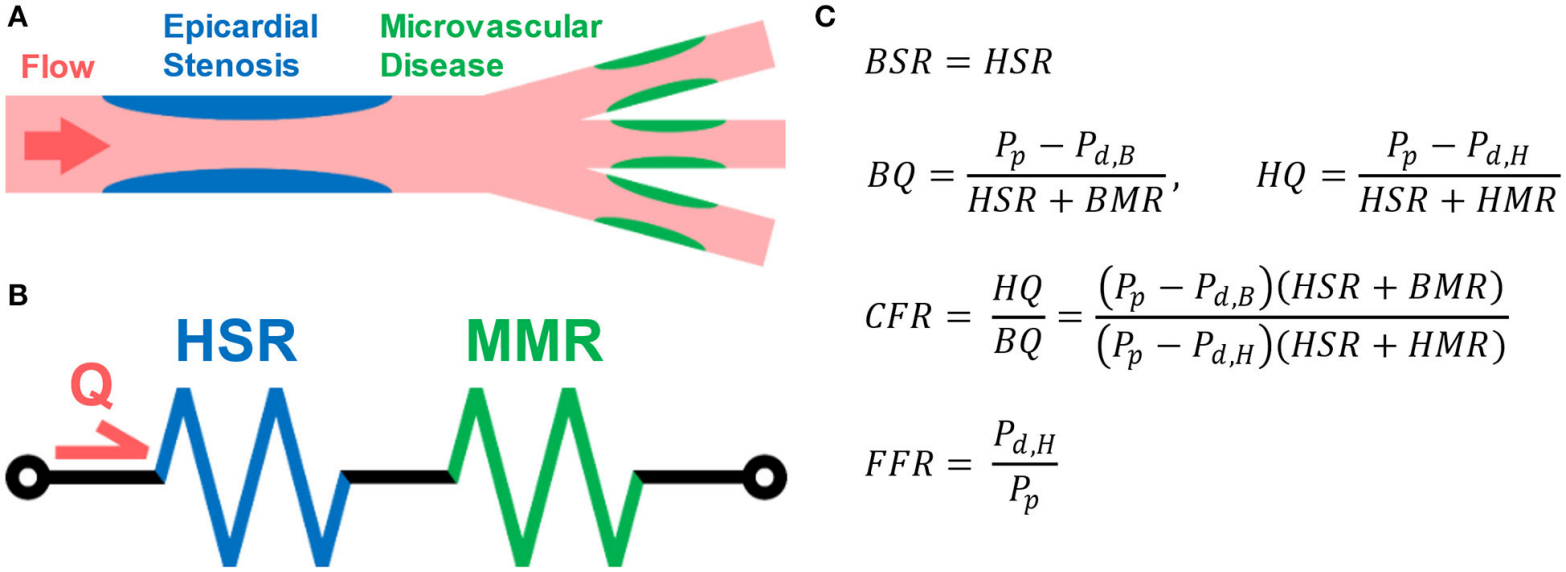
Illustration of Coronary Flow Reserve (CFR). **(A)** Coronary arterial blood flow travels through both epicardial vessels and microvasculature. Each can contribute to the total resistance. The resistance due to the epicardial vessels (blue) is the stenotic resistance (SR), and the resistance due to the microvasculature (green) is the myocardial microvascular resistance (MMR). **(B)** The flow can be modeled using a circuit analogy in which the total resistance is the sum of the SR and MR. **(C)** MR during basal (BMR) and hyperemic (HMR) flow conditions should vary, but SR should remain the same in both (HSR). The basal flow rate (BQ) can be estimated as the difference in the proximal pressure (P_p_) and distal, basal pressure (P_d,B_) divided by the total basal resistance. Similarly, the hyperemic flow rate (HQ) can be estimated using the distal, hyperemic pressure (P_d,H_) and total hyperemic resistance. CFR is defined as the ratio of HQ to BQ, while FFR is the ratio of P_d,H_ to P_p_. Through these equations, the theoretical interdependence of CFR and FFR can be seen.

A complimentary coronary physiologic measure to FFR is coronary flow reserve (CFR), which is defined as the ratio of hyperemic flow to basal flow (8). In healthy individuals, CFR has been shown to be ~4.8, which indicates hyperemic flow is almost five times greater than basal flow (8, 9). Unlike FFR, CFR is affected by both epicardial vessel resistance due to stenoses and distal tissue bed vascular function (10). Because CFR is affected by total vascular resistance and FFR only reflects epicardial vessel resistance, *CFR value can vary substantially between patients with the same FFR values* (Figure 1) (10–13). CFR can be measured clinically through magnetic resonance imaging (MRI) and positron emission tomography (PET) and can be estimated through single-proton emission computed tomography (SPECT).

Virtual fractional flow reserve (vFFR) combines non-invasive coronary imaging with computational fluid dynamics (CFD) to estimate FFR. To compute vFFR, certain boundary conditions must be defined in the patient-specific model, including: the lateral wall geometry which describes the coronary luminal boundary; the inlet flow rate to simulate hyperemic coronary blood flow; and flow-splitting ratios at vessel branch points. Computed tomography (CT) has proven to be an attractive modality for defining these boundary conditions due to its excellent spatial resolution which can characterize the coronary arterial geometry (14). However, it cannot quantify the other boundary conditions directly due to its inability to measure flow. With CT-derived vFFR (FFR_CT_), the total myocardial mass of the individual can be estimated from the CT image data, which allows for an estimation of the patient-specific basal coronary arterial flow, or the flow through the coronary arteries when the subject is at *rest*, through allometric scaling (14). The rationale behind this is that the rate of myocardial blood flow should be proportionate to the amount of myocardial tissue (15). This relationship, however, only applies to *basal* coronary flow, while FFR is defined only during hyperemic, or stress, flow conditions.

To account for this requirement of hyperemic flow, the predicted basal flow must be artificially scaled by an estimated CFR value, which has been done through direct modification of the resistances within the model (14). The epicardial resistance is automatically adjusted through the presence of a stenosis, but CFR is determined by both the epicardial and microvascular responses to stress, and CT has no means through which it can estimate patient-specific microvascular resistance. Therefore, the microvascular resistance must be scaled using a *population-average* response rather than a *patient-specific* one (14). Because FFR quantifies the pressure drop across the stenotic lesion and the pressure gradient is directly related to the flow rate *via* Ohm's Law, it follows that any linear change in the inlet flow would likely result in a proportionate change in the calculated vFFR. *Therefore, it is hypothesized that basal flow scaled by patient-non-specific CFR cannot accurately calculate vFFR*.

The purpose of this study is to investigate the effect of varying CFR values on the calculated vFFR value. A coronary geometry exhibiting an intermediate stenosis was acquired through magnetic resonance angiography (MRA) and produced a constant epicardial resistance for a series of computational simulations. Basal and hyperemic (under adenosine administration) coronary flow waveforms were acquired in a series of 13 patients using phase-contrast MRI (PCMR) which enabled calculation of CFR. Flow through the coronary geometry was simulated at a range of hyperemic flow conditions determined by the measured CFR values and enabled determination of the resulting vFFR.

## Methods

### Overview of Methodology

**Figure 2:**
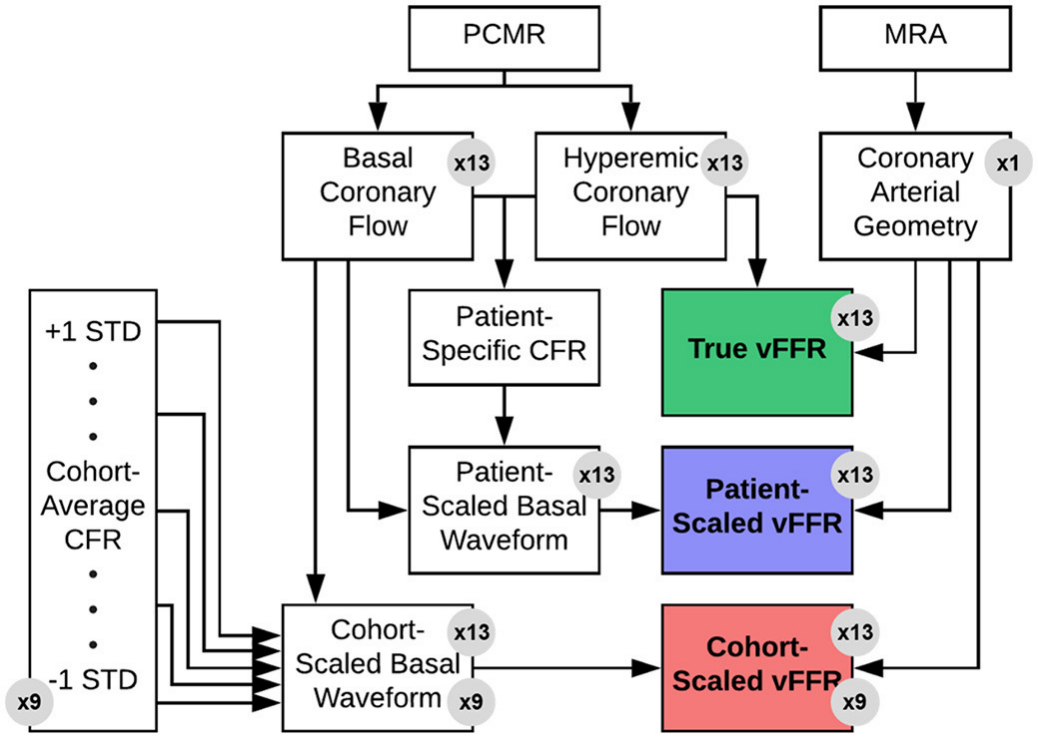
Overview of experimental design. A single left coronary artery tree was acquired from an MRA of a patient with a 50% stenosis in the LAD. Hyperemic and basal flow waveforms were measured in patients (*n* = 13) using PCMR. Each hyperemic flow waveform was applied as an inflow boundary conditions for the anatomic model to determine the true vFFR value *via* CFD. Each basal flow waveform was then scaled by either the patient-specific or one of nine estimated CFR value to approximate the hyperemic flow. These scaled flow rates were then applied as inflow boundary CFD simulations to estimate vFFR—i.e., patient-scaled vFFR or cohort-scaled vFFR. These scaled vFFR values were then compared to the corresponding true vFFR values.

An overview of the experimental approach is presented below, followed by a detailed explanation of each component of the methodology. First, a coronary arterial computational model was constructed using MRA image data acquired from a patient presenting with moderate CAD. Second, resting and hyperemic time-dependent flows through the coronary sinus were measured in a separate cohort of patients (*n* = 13) undergoing clinically indicated stress cardiovascular MRI exam. Third, CFD was used to determine vFFR values with various applied hyperemic flow conditions, including: (i) *in vivo* hyperemic flow by PCMR measurement (true vFFR), (ii) basal flow scaled by the patient-specific CFR (patient-scaled vFFR), and (iii) basal flow scaled by population-average CFR estimates (cohort-scaled vFFR). These scaled vFFR values were compared with the true vFFR values to assess correlation and concordance, thereby evaluating how changing the hyperemic flow response—as measured by CFR—as well as time-dependent flow patterns affect vFFR prediction with a constant geometry. A graphical flowchart of these methods can be seen in Figure 2. The study was approved by the university's Institutional Review Board.

*The purpose of this study was to investigate the effects of flow conditions on vFFR, not specifically to validate vFFR measurements against an invasive gold standard*. Because the variable of interest in this study is the simulated hyperemic flow, the arterial geometry was maintained as a constant to isolate the effect of the flow behavior. Therefore, the calculated vFFR values are not intended to be representative of any particular subject's true functional significance; rather, they are only meant to be compared against other non-invasive estimates to see how the predicted values change with variable flow.

### Coronary Anatomy Model Geometry

**Figure 3:**
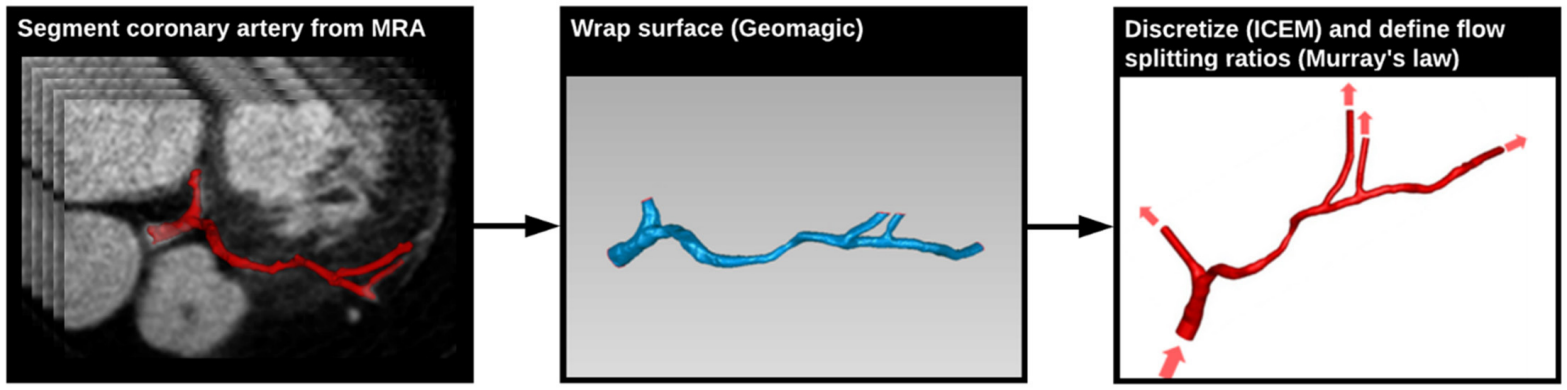
Generation of coronary arterial geometry from MRA data. The left main (LM), left anterior descending (LAD), and left circumflex (LCX) arteries were segmented from a 3D MRA dataset data using the Vascular Modeling Toolkit (VMTK) **(left)**. The resulting surface geometry was imported into Geomagic to generate a smooth 3D surface **(center)**. This surface was imported into ICEM meshing software to generate the 3D computational mesh, and transient CFD simulations were run using Fluent **(right)**. Flow boundary conditions were based on PCMR measured flows.

A patient presenting with NYHA class III ischemic heart failure was imaged prior to cardiac resynchronization therapy as part of an IRB-approved study (16). Imaging was performed on a 3 T MRI scanner (MAGNETOM Trio, Siemens Healthcare) using a six-element phased-array cardiac coil. A 3D, whole-heart, navigator- and ECG-gated inversion-recovery FLASH sequence with a centric *k*-space trajectory acquired coronary images in the transverse plane at a resolution of 0.64 × 0.64 × 0.75 mm^3^. Images were acquired in diastole during the slow infusion of a gadolinium-based contrast agent (17). The left main (LM), left anterior descending (LAD), and left circumflex (LCX) arteries were segmented from the image data using a Frangi vessel enhancing post-processing filter followed by a colliding fronts segmentation algorithm (Vascular Modeling Toolkit) (18–20). The resulting triangulated surface geometry was imported into Geomagic (Geomagic, Inc.) to generate a smooth 3D surface. This surface was imported into ICEM meshing software (ANSYS, Inc.) to generate the 3D computational mesh. Flow extensions were added to the inlet and each outlet by projecting the edge contour in line with the local trajectory of the boundary surface (Figure 3). The model was generated with ~100,000 tetrahedral elements and 150,000 six-node pentahedral elements comprising eight boundary layers with a linear growth factor of 1.1 such that each innermost element was approximately the same volume as the adjacent tetrahedral element. Previously analysis of patient-specific models of epicardial coronary vessels has demonstrated solution independence at this mesh density (21).

### Coronary Flow Measurements

**Figure 4:**
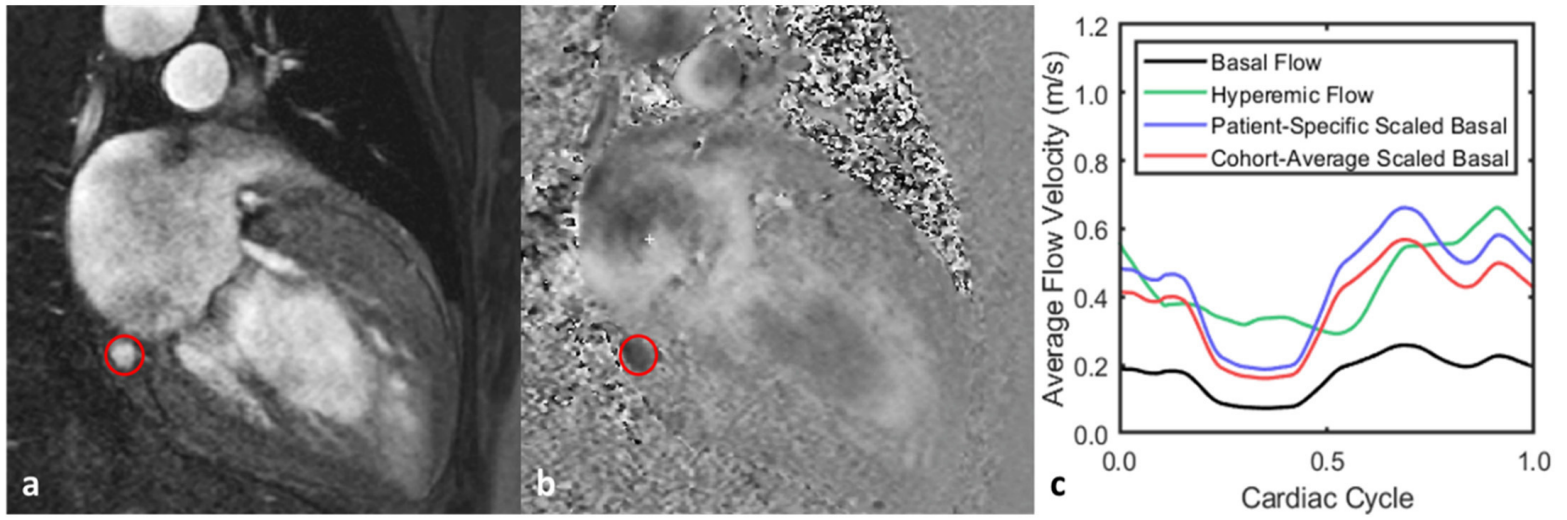
Representative flow waveform measurement for one patient. **(a,b)** Magnitude and phase images acquired from PCMR acquisition with the coronary sinus outlined. **(c)** Both the basal and hyperemic flows were acquired using PCMR, and the basal flow was then scaled by a range of CFR values. When the basal flow is scaled by the patient-specific CFR—2.8—, the time-average flow rate is the same for both it and the hyperemic flow. Scaling the basal flow by the cohort-average CFR—2.2—produces the same basal waveform but with a different time-average flow rate from the hyperemic flow.

Coronary sinus flow measurements were acquired in patients (*n* = 13) who had been clinically indicated for a cardiac stress MRI at Emory University Hospital on a 1.5 T scanner (MAGNETOM Avanto^fit^, Siemens Healthcare) using a twenty-element phased-array cardiac coil (22–24). As part of the routine scan, a low-resolution axial 3D volume was first acquired for planning purposes. Multiplanar reformation of this volume determined a plane which perpendicularly intersected the proximal coronary sinus immediately adjacent to its ostium into the right atrium (Figures 4A,B). An ECG-gated, 2D PCMR cine was acquired on this plane during a breath-hold with a field-of-view of 350 × 306 mm, an in-plane pixel spacing of 0.68 mm, and a slice thickness of 6 mm. The velocity-encoding value (VENC) in each scan was initially set at 60 cm · s^−1^ and adjusted if aliasing was observed in the velocity-encoded image. Each patient was imaged once during a resting state and again following a 3 min infusion of intravenous adenosine at a rate of 140 μg · kg^−1^ · min^−1^ to induce maximal coronary hyperemia.

Following data acquisition, images were exported offline and analyzed using the freely available software, Segment version 2.0 (25). For each image stack, the magnitude and phase images were coupled and used to identify the luminal contours of the target vessel. A constant region-of-interest (ROI) was used across each temporal phase, and the ROI size was maintained when evaluating repeated measurements of the same vessel. Static tissue regions at the chest walls were automatically identified and used to calculate a second-order polynomial map to represent the estimated phase error and correct for this (25). The through-plane velocities of the pixels contained within the ROI were then added to provide a time-dependent flow waveform. This procedure was repeated for images acquired both during rest and during stress. Based on previously reported measurements of coronary arterial flow rates (26–28), the left coronary arterial flow rate was assumed to comprise ~2 thirds of the total coronary flow. The measured coronary sinus flow waveforms were then scaled by this value to give an estimation of the inflow waveform of the LM.

### Hyperemic Flow Conditions for CFD Simulations

Various hyperemic flow conditions were applied to evaluate their effects on computed vFFR values. The first condition was the pulsatile hyperemic flow measured *in vivo* for each patient, which was used to define the true vFFR against which the other predicted vFFR values would be compared. Next, the patient-specific CFR value was calculated by taking the ratio of time-averaged, hyperemic-to-basal flow rates across the cardiac cycle. For each patient, the measured basal flow was scaled by the patient-specific CFR value to give a flow waveform with the same time-averaged flow rate as the measured hyperemic flow (Figure 4). This flow was used to compute the patient-scaled vFFR. Lastly, the basal flow rate was scaled by a series of global estimates of the CFR which were not specific to the patient but were representative of the cohort as a whole. In total, nine patient-non-specific CFR values were used to cover the range of one standard deviation above and below the cohort average value. Each of these computed waveforms were used to compute a cohort-scaled vFFR value for the patients.

### CFD and vFFR Calculation

Transient (i.e., pulsatile) CFD simulations were run using Fluent (ANSYS, Inc.). There were 13 patients on whom coronary flow measurements were obtained, and for each of these patients 11 vFFR values were computed: the true vFFR found using the actual hyperemic flow waveform, the patient-scaled vFFR found by scaling the basal waveform with the patient-specific CFR, and nine cohort-scaled vFFR values found using the patient-non-specific CFR estimates and the basal waveform. These patient-non-specific CFR estimates were constant across the entire cohort.

For each case, the simulated hyperemic flow rate was prescribed as a time-varying blunt inlet flow boundary condition, inlet pressure was set at 100 mmHg, and mass flow splits, which were derived from Murrays law, were applied at each outlet flow surface (29). The transient solution was computed across three pulsatile cardiac cycles comprising 300 time steps, each at a heart rate of 60 beats per minute. Blood was modeled as Newtonian with a density of 1,060 kg · m^−3^ and dynamic viscosity of 0.0035 Pa · s. Because the PCMR flow measurements were acquired across only 18 temporal phases, the waveforms were resampled to 300 time steps using a first order linear interpolation scheme followed by a lowpass filter to create a smooth waveform with the same time-averaged flow rate. We used the SIMPLE algorithm for pressure-velocity coupling and second-order Green-Gauss node-based discretization for momentum and pressure. For each time step, convergence was achieved once the residuals of momentum and continuity fell below 10^−5^. The computed pressure was then sampled along the centerline of the vessel and divided by the inlet pressure to calculate vFFR along the length of the vessel, in accordance with clinical practice. The clinically relevant vFFR value—found 4 mm distal to the region of minimal lumen area—was then time averaged and recorded.

### Statistical Analysis

Across all tested CFR values—both patient-specific and patient-non-specific—, the error of the computed scaled vFFR was calculated in relation to the true vFFR found with the corresponding hyperemic waveform. Within each simulated CFR group, a two-tailed, paired *t*-test was used to evaluate that error at a significance level of 0.05. Correlation between each scaled vFFR value and its corresponding true vFFR value was calculated through the Pearson correlation coefficient, and concordance was evaluated through a Bland–Altman analysis and calculation of Lin's concordance correlation coefficient (30, 31).

## Results

**Figure 5:**
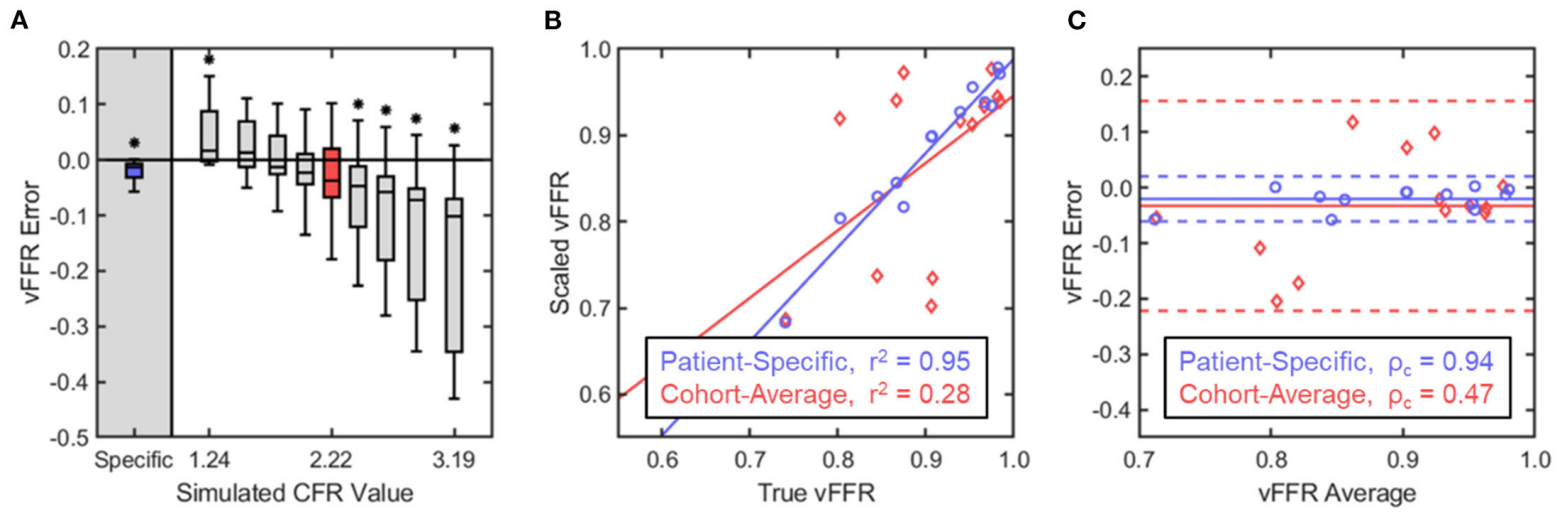
Statistical analysis of vFFR estimation with variable flow conditions. **(A)** vFFR error was defined as the difference in the scaled vFFR value calculated with the basal waveform and the true vFFR value calculated with the hyperemic waveform. Within each group, the boxes represent the interquartile range and median while the whiskers indicate the 10 and 90th percentiles. Comparison within each simulated CFR group against the true vFFR was performed through a paired, two-way *t*-test, with significance determined for *p* < 0.05. **(B)** Scaled vFFR values were plotted against the corresponding true vFFR values for two CFR groups: (1) the patient-specific CFR values and (2) the cohort-average CFR value of 2.2. Linear regression determined a line of best-fit for each group, and Pearson's correlation coefficient determined the linearity of the relationship. **(C)** A Bland–Altman analysis and calculation of Lin's concordance correlation coefficient was performed to assess the concordance of the relationship. For each match-pair of true vFFR and scaled vFFR, the difference between the two values was plotted against their average. The solid lines indicate the mean vFFR error while the dashed lines indicate two standard deviations from the mean.

The CFR values for the cohort ranged from 1.2 to 4.1 with a mean value of 2.2 ± 0.98. The difference between the various scaled vFFR values and the true vFFR (termed vFFR error) was determined across all 13 patients. Paired comparison of the patient-specific vFFR with the true vFFR produced a mean vFFR error of −0.02 ± 0.02 with a *p*-value of 0.004; scaling the basal flow waveforms by the cohort-average CFR of 2.2, however, gave an average error of −0.03 ± 0.1 and a *p*-value of 0.236 (Figure 5A). Mean vFFR error was seen to monotonically decrease with increasing simulated CFR value, and, in general, the distribution of the error was seen to be larger with deviation from the cohort-average CFR, and increasing significance was observed on either end of the range of tested values (Figure 5A). The patient-specific vFFR produced the smallest error variance with the true vFFR, and their relationship was seen to be strongly linear; conversely, the cohort-average vFFR showed a very weak linearity when plotted against true vFFR (Figure 5B). None of the patient-non-specific CFR values were able to produce a coefficient of determination >0.34 (Table 1). Similarly, the patient-specific vFFR showed a strong concordance with true vFFR, while the cohort-average showed a very weak concordance (Figure 5C). Across all patient-non-specific CFR values simulated, the concordance correlation coefficient was seen to be between 0.14 and 0.34 (Table 1).

**Table 1:** Statistical results for all simulated CFR values.

	**Patient-specific**					**Cohort-average**				
CFR		1.24	1.54	1.78	2.01	***2.22***	2.43	2.66	2.89	3.19
*p*-value	**0.004**	0.019	0.191	0.883	0.400	**0.236**	0.043	0.018	0.009	0.005
Pearson's *r*^2^	**0.95**	0.34	0.34	0.34	0.34	**0.28**	0.34	0.34	0.34	0.34
Lin's *ρ_*c*_*	**0.91**	0.21	0.31	0.34	0.33	**0.25**	0.25	0.21	0.17	0.14

*Patient-specific and Cohort-average columns are bold for emphasis*.

## Discussion

The major findings of this study are: (1) scaling the basal coronary flow waveform by a patient-non-specific CFR estimate *cannot* provide predictive results for all individuals within a cohort, and (2) using the patient-specific CFR to scale the basal waveform yields vFFR values which are strongly predictive of those found with the hyperemic flow waveform.

Myocardial ischemia can result from epicardial disease, microvascular disease, or a combination of both. Clinical indication for intervention in epicardial stenosis has been shown to be most successful when guided by the FFR, which approximates the reduction in flow through a given vessel due to an anatomic narrowing. Accurate measurement of this flow reduction relies on the assumption of maximal hyperemia, in which the downstream microvascular resistance is minimized. The physiologic response to hyperemia is, however, patient-specific, and depends on both the stenotic resistance contributed by the epicardial vessels and the downstream hyperemic microvascular resistance. Though it is possible to estimate the value of hyperemic stenotic resistance (HSR) through anatomic measurements and allometric scaling, the hyperemic myocardial resistance (HMR) can only be measured through direct quantification of flow (10). The comorbidity of both epicardial and microvascular coronary disease is not uncommon; however, the presence or severity of one cannot be used as a direct indicator of the presence or severity of the other. In 2017 it was shown that as few as 68% of patients with moderate coronary stenosis had concordant FFR and CFR findings (10).

vFFR is an emerging methodology that seeks to provide a non-invasive alternative to invasive catheter-based FFR. Arguably the most well-known of these approaches is FFR_CT_ (14). As CT is incapable of quantifying the CFR or HMR, its flow boundary conditions rely on population-average physiologic responses to hyperemia. Though results of this methodology for vFFR have shown some success in predicting invasive FFR, the correlation between the two measurements does not indicate strong correlation (*r*^2^ = 0.54), and its diagnostic accuracy has shown to suffer substantially when predicting FFR values near the clinical cutoff (0.80) where specificity is most needed by clinicians (32). It is possible that these limitations in efficacy are due at least in part to the assumptions made regarding HMR and, by extension, CFR.

Scaling the basal flow waveform by the time-averaged, patient-specific CFR does not replicate the exact hyperemic time-dependent flow waveform for a given patient. This is due to the interactions between the myocardium and the microvasculature, resulting in phasic fluctuations of the intracoronary resistance (33). Since the intracoronary resistance is not uniform across the cardiac cycle during resting flow, it has varying levels of response to hyperemic induction as well, resulting in a time-dependent CFR (Figure 4C). However, because FFR is calculated as the time-averaged ratio of distal and proximal pressure in the coronary arteries, it was expected to be insensitive to temporal fluctuations and depend only on the time-averaged flow rate and epicardial anatomy. By scaling the basal flow waveform by the patient-specific CFR, the resulting vFFR values were seen to show very strong correlation and concordance with those calculated using the hyperemic waveform, which would indicate support for this hypothesis. However, the significant paired differences between the two groups does suggest that these waveforms may not be completely interchangeable for all populations. In general, across all CFR values tested, we observed an underestimation for vFFR when using the basal waveforms from the patients, which would indicate that differences in the temporal behavior between these two waveforms can result in significantly different vFFR calculations. Though the concordance and correlation values for this cohort were high, these results suggest that vFFR is not completely insensitive to time-dependent behaviors, and therefore more thorough testing should be performed to investigate which populations can interchangeably use a basal or hyperemic waveform for the calculation of vFFR.

Scaling the basal flow by some non-patient-specific CFR values near the population average produced vFFR values that were, on average, not significantly different from hyperemic vFFR, but deviation from the population average resulted in increasingly large deviations in estimating vFFR. Predictive power of CFR-scaled vFFR for hyperemic vFFR was never strong for any estimated CFR value, and correlation was mostly independent of the estimated CFR as well. This indicates that patient-specific characterization of the hyperemic flow rate needs to be used to accurately predict vFFR. Therefore, the choice in imaging modalities is limited to those that can measure both anatomy and flow. One could use a combination of imaging modalities to accomplish this, such as using both CT—to acquire the coronary anatomy and estimate the basal coronary flow rate through allometric scaling as described by Choy and Kassab (15)—and positron emission tomography (PET)—to assess the patient-specific CFR and determine the hyperemic response (34). Perhaps a more feasible clinical solution would be to use MRI which can acquire both anatomy and flow directly (35, 36). The use of PCMR to directly estimate the pressure gradient across a coronary stenosis has also been shown to be feasible, which has the potential to eliminate the need for CFD flow simulations altogether (37).

There are some limitations to this study. All comparisons were made between artificial vFFR values without direct comparison with an invasive FFR measure. As was stated previously, such a comparison would not be valid for this study, as the coronary geometry was maintained as a constant so that the effects of flow variation could be isolated. The results presented here strongly indicate that calculating vFFR without patient-specific hyperemic flow conditions can produce inaccurate results. It is possible that other sources of error may exist estimating FFR through vFFR, including inaccuracies within the coronary geometry or the outflow boundary conditions. Additional studies to test each of these variables would be needed to draw such conclusions. It is acknowledged that blood is a non-Newtonian fluid; however, the Reynolds (Re) numbers in these computational models were moderate (Re ≈ 250–400), and the impact of the non-Newtonian behavior of blood on the hemodynamic environment is minimal (38). Finally, the computational models assumed a rigid coronary wall. We acknowledge that the presented models only approximate the *in vivo* conditions, and that application of a validated fluid-structure interaction (FSI) computational framework may provide improve accuracy in the predicted hemodynamics measures.

The coronary inlet flow rates used within this study were not directly measured from the patients' coronary arteries but were linearly scaled waveforms acquired from the coronary sinuses. This approximation was used as direct arterial flow measurement through PCMR could not be feasibly integrated into the clinical protocol. Though the arterial flow and the coronary sinus flow rates do not exhibit identical temporal behaviors, the functional behavior of one should give an indication of the other due to their similarities. Due to the aforementioned suspected temporal dependence of vFFR, it is possible that this approximation may have resulted in the patient-specific scaled vFFR group performing better or worse than the equivalent arterial flow waveform would have in estimating the true vFFR; however, this approximation should not undercut the conclusion that using patient-non-specific hyperemic response cannot give a strongly predictive estimation of the true vFFR.

This study found that scaling the basal flow waveform by an estimated patient-non-specific CFR will not accurately predict the vFFR calculated using the actual hyperemic flow waveform; using the patient-specific CFR, however, was seen to provide more consistent and accurate measurements of vFFR. The necessity for having patient-specific hyperemic flow behavior means that assumptions about CFR cannot be used to scale the patient's basal flow prior to its use as a boundary condition without significantly sacrificing the model's predictive power. It is recommended to execute additional studies to identify the extent to which the temporal behavior affects the computed vFFR.

## Data Availability Statement

The raw data supporting the conclusions of this article will be made available by the authors, without undue reservation.

## Ethics Statement

The studies involving human participants were reviewed and approved by Emory University Institutional Review Board. The patients/participants provided their written informed consent to participate in this study.

## Author Contributions

JH and JO developed the hypothesis and planned the methodology. JH oversaw the image acquisition and constructed the vessel geometry. RE performed the computational simulations with oversight from JH and LT. HS provided clinical perspectives to help inform the discussion. All authors reviewed and approved the final manuscript.

## Conflict of Interest

The authors declare that the research was conducted in the absence of any commercial or financial relationships that could be construed as a potential conflict of interest.
